# Harnessing Mechanisms of Immune Tolerance to Improve Outcomes in Solid Organ Transplantation: A Review

**DOI:** 10.3389/fimmu.2021.688460

**Published:** 2021-06-10

**Authors:** Priscila Ferreira Slepicka, Mahboubeh Yazdanifar, Alice Bertaina

**Affiliations:** Division of Hematology, Oncology and Stem Cell Transplantation and Regenerative Medicine, Department of Pediatrics, Stanford University School of Medicine, Stanford, CA, United States

**Keywords:** immune tolerance, hematopoietic stem cell transplantation, solid organ transplantation, innate immunity, adaptive immunity

## Abstract

Survival after solid organ transplantation (SOT) is limited by chronic rejection as well as the need for lifelong immunosuppression and its associated toxicities. Several preclinical and clinical studies have tested methods designed to induce transplantation tolerance without lifelong immune suppression. The limited success of these strategies has led to the development of clinical protocols that combine SOT with other approaches, such as allogeneic hematopoietic stem cell transplantation (HSCT). HSCT prior to SOT facilitates engraftment of donor cells that can drive immune tolerance. Recent innovations in graft manipulation strategies and post-HSCT immune therapy provide further advances in promoting tolerance and improving clinical outcomes. In this review, we discuss conventional and unconventional immunological mechanisms underlying the development of immune tolerance in SOT recipients and how they can inform clinical advances. Specifically, we review the most recent mechanistic studies elucidating which immune regulatory cells dampen cytotoxic immune reactivity while fostering a tolerogenic environment. We further discuss how this understanding of regulatory cells can shape graft engineering and other therapeutic strategies to improve long-term outcomes for patients receiving HSCT and SOT.

## Introduction

Solid organ transplantation is a lifesaving therapeutic strategy for numerous end-stage organ failures. The past 25 years have witnessed undeniable progress in preventing graft rejection and graft-versus-host-disease (GvHD), but these gains rely on lifelong use of immune suppressive (IS) drugs (1). Long-term IS regimens contribute to poor clinical outcomes by leading to severe side effects including cardiovascular diseases, hypertension, diabetes, nephrotoxicity, and an increased risk of cancer and infections (2). Even with IS drugs, graft loss occurs in half of patients within 15 years for histocompatibility leukocyte antigen (HLA)-mismatch kidney transplant recipients and within 25 years for those who are fully HLA-matched (3). Currently, many children who receive a SOT at a young age will need at least one additional transplant during their lifetime because of inevitable loss of their graft caused by the combination of chronic rejection, infections, drug toxicity, and nonadherence (4).

Despite great advances in the induction of tolerogenesis in humanized mouse and classical preclinical models (5), there is still a large gap in translating this success to the bedside. Spontaneous operational tolerance remains rare, occurring in less than 5% of kidney and 20% of liver transplant recipients (6–9). Studies have found that some patients who have persistent graft acceptance with chronic IS drug use can become tolerant, allowing careful reduction and eventually full cessation of IS treatment. However, given current challenges in identifying biomarkers of graft rejection, removing IS—especially after kidney transplants—is risky and can lead to graft loss with consequent reductions in life expectancy (10–12). Enhancing long-term outcomes for patients of all ages requires new approaches to transplantation that can address these challenges.

In recent decades, investigators have focused on developing alternative approaches to induce immune tolerance toward the donor graft in transplant recipients. A standout example is the combination of allogeneic HLA-matched HSCT with SOT from the same donor (13). Despite promising results, over 70% of patients lack an HLA-identical sibling. For this reason, transplants from related full-haplotype mismatched (haploidentical) donors (14) and unrelated HLA-matched and mismatched donors have been performed to expand availability of this treatment protocol.

Successful allogeneic HSCT requires the development of immune tolerance towards both the donor and host allogeneic antigens. Induction of immune tolerance can prevent T-cell mediated graft-rejection and GvHD, which might lead to life threatening complications in HSCT recipients. Current approaches to prevent rejection and GvHD after HSCT primarily rely on pharmacological IS, either prior to or after HSCT. These approaches are limited by lack of antigen specificity, and the requirement for long-term therapy, which often leads to severe complications. Recent progress in understanding the mechanism of action of alloreactive and regulatory cell populations has led to the use of specific cell subsets to prevent/treat graft rejection and GvHD and induce immune tolerance. Peripheral tolerance after allogeneic HSCT may be achieved by several mechanisms, though blocking alloreactivity to the host human leukocyte antigens while preserving immune responses to pathogens and tumor antigens remains a challenge. Recently uncovered evidence regarding the mechanisms of post-HSCT immune reconstitution and tolerance in transplanted patients has allowed for the development of novel cell-based therapeutic approaches. These therapies are aimed at inducing long-term peripheral tolerance and reducing the risk GvHD, while sparing the graft-versus-leukemia (GvL) effect (15).

The use of sequential HSCT and SOT has resulted in meaningful improvements in kidney graft tolerance (16–24). With the addition of non-myeloablative conditioning, many HLA-matched recipients are able to taper and fully discontinue all IS drugs within two years after transplantation without GvHD or graft rejection (25). In HLA-mismatched recipients, though, achieving tolerance without IS has proven to be considerably more difficult and, when accomplished, has often come with heightened risks of GvHD and infections that can threaten graft survival (25–28). Although clinical studies have made strides in maintaining long-term organ engraftment with reduced IS regimens, there is an ongoing need to improve immune tolerance after sequential HSCT and SOT in order to completely eliminate the need for pharmacological IS and without potentially risky tradeoffs for patient outcomes.

A refined understanding of the mechanisms of immune tolerance creates opportunities for novel HSCT techniques- including graft engineering strategies- to optimize survival after SOT and enable a functional immune system that permanently accepts donor antigens without the need for IS. This review describes first key findings that influence our understanding of the cellular mechanisms involved in immune tolerance as well as the role of innate immunity in these regulatory processes. It then explores how development of new therapeutic strategies can harness this knowledge to more effectively induce tolerance, especially in the context of sequential HSCT and SOT.

## Cellular Mechanisms of *Conventional* Immune Tolerance

Immune tolerance is multifaceted and involves the interaction of different cells, listed in **Table 1**, that serve critical regulatory roles. While investigators have long worked to identify processes of tolerogenesis, advanced methods, including single-cell technologies, have expanded the mechanistic understanding of cells that are actively involved in the development of immune tolerance.

**Table 1:** Features of conventional and unconventional immune regulatory cells.

Cell type (Cell surface/intracellular markers)	Plasticity (Cell surface/intracellular markers)	Signaling factors to induce plasticity	Homing (Cell surface markers)	References
Tregs (CD4^+^CD25^+^ CD127^-/lo^	Th1-like Tregs (IFN-γ^+^/T-bet^+^/CXCR3^+^)	Th1-like: IFN-*γ*, IL-12, IL-27, IL-4, TGF-β, IL-2	Gut (GPR-15)	(29–35)
Naïve Tregs (CD45RA^+^FoxP3^+^)	Th2-like Tregs (IL-4^+^/IL-5^+^/IL-13^+^/GATA3^+^)	Th2-like: IL-4; IL-5	Inflammation areas (CXCR3, LFA-1, VLA-4, CCR2, CCR5, CCR6, CCR8)	
*FOXP3* ^+^ effector non-Tregs (CD45RA^-^ FoxP3^low^)	Th17-like Treg (IL-17A^+^/RORγt^+^)	Th17-like: IL-6, IL-21, IL-12, IL-23, TGF-β, IL-2, GATA3, IDO	Secondary lymphoid organs (CCR7, CD62L)	
Non-classic Tregs (CD4^+^CD25^+^CD5^+^CD38^−/lo^CD45RA^+^)	Follicular regulatory T cells -Tfr (CXCR5^+^/Bcl6^+^/ICOS^+^/PD1^+^)	Tfr: IL-6, IL-21	Skin (CCR4)	
Activated/effector Tregs (CD25^hi^CD127^lo^CD45RO^+^ CD45RA^-^FoxP3^high^)				(36–38)
Tr1 (CD4^+^ CD49b^+^ LAG-3^+^ CD226^+^)	Tr1 can be derived from	Th1 (TCR signaling, CXCL12, IL-12, IL-27)	Gut (GPR15, CCR9 – *in vitro* induced Tr1)	(39–43)
	Th1, Th2, Memory CD4+ T cell and Th17	Th2 (TCR signaling)	Spleen (unknown)	
		Memory CD4+ T cells (TCR signaling)		
		Th17 (IL-27, TGF-β)		
Bregs -Transitional (CD19^+^CD20^+^CD10^+^ CD27^-^CD24^high^CD38^high^)	Possible high plasticity	–	Inflamed skin	(44–46)
Bregs -Transitional TIM-1^+^ (CD19^+^ CD24^high^ CD38^high^ TIM-1^+^)				
Bregs - Memory/Mature (CD19^+^CD20^+^CD10^-^CD27^+^ CD24^high^CD38^-^)				
γδTregs (CD25^low^ CTLA-4^low^	Unknown for γδTregs	Th1-, Th2-like (pAg, IL-2, IL-4)	Kidney, Liver, Lung, Intestine (V*γ*1, V*γ*3, V*γ*5)	(47–54)
CD8^+^ - mouse renal allografts)	Vδ2 – High plasticity (Th1-, Th2-, Th9-, Th17-, Tfh-like cells)	Th9-like (IL15, TGF-β)	Gut (CD103, α4β7)	
		Th17-like (pAg, Il-6, IL1g, TGF-β)		
		Tfh-like (pAg, IL-21)		
Induced γδTregs (FoxP3^+^)				
NKT (CD161^+^ TCR Vα24Jα18^+^ PLZF^+^)	NKT1 (PLZF^lo^, T-bet^high^, IFN-*γ* ^high^)	–	Liver (CXCR3, CXCR4)	(55–57)
	NKT2 (PLZF^high^, T-bet^low^, IL-4)		Lung (CCR4)	
	NKT17(PLZF^+^, ROR*γ*t^+^/, IL-17)		Spleen (CCR7, CXCR3-6)	
NKregs (CD56^bright^ CD16^-/low^ NKp46^+^	Unknown		unknown	(58, 59)
Granzyme B^low^ Perforin^low^)				

### ‘Conventional’ Treg Cells

In 1970, a seminal study by Gershon and Kondo (60) described a subset of T cells distinct from T helper (Th) cells that decreased the immune response. Twenty-five years later, these cells were named regulatory T cells (Tregs) in a study that found athymic mice inoculated with purified CD4^+^CD25^-^ T cells spontaneously developed autoimmune diseases (61) whereas the transfer of CD4^+^CD25^+^ cells inhibited CD4-mediated autoimmunity in lymphopenic mice.

The ontogeny of naturally emerged Tregs occurs in the thymus (tTregs) while other Tregs are converted or induced from CD4^+^CD25^-^ in the periphery (iTregs or pTregs, respectively) (62). tTregs are crucial for control of immune self-tolerance, allergy, and allograft survival. In mice and humans, tTregs comprise 2-10% of peripheral CD4^+^ T cells (63, 64). Interleukin-2 (IL-2)-receptor α chain (CD25) is a cell surface marker that identifies Treg cells. Stimulation with TNF tumor necrosis factor (TNF) and IL-2 upregulates CD25 and activates Tregs (65); however, induction of CD25 expression in CD25^-^ murine T cells is not sufficient to generate Treg suppressive function (66). Notably, activated memory and certain effector T cells (Teff) can also express CD25 (66). Thus, phenotype subsets of Tregs have been more precisely identified with other cell surface markers.

The identification of Forkhead Box P3 (*FOXP3)* as a master regulator of the Treg lineage commitment and differentiation has dramatically improved understanding of Treg biology (67–69). Loss-of-function mutations in human *FOXP3* cause Immunodysregulation Polyendocrinopathy Enteropathy X-linked (IPEX) syndrome, a rare and life-threatening immune disease (70). *FOXP3* mutation or deletion can also lead to loss of repression of oncogenes in some nonlymphoid cells, resulting in malignancies (71, 72). Early onset IPEX syndrome exclusively affects males and leads to fatal lymphoproliferative dysfunction in Tregs and subsequent severe autoimmunity (70, 73). The Treg specific demethylation of a highly conserved non-coding element within the *FOXP3* gene (Treg-specific demethylated region, TSDR) is required for *FOXP3* expression and can be used for Treg identification (74, 75). However, TSDR methylation status can vary; it is fully demethylated in tTregs, partially methylated in TGF-β polarized Tregs, and methylated in naïve cells (75–77). Accordingly, the methylation status of the *FOXP3* TSDR is a marker for the stability of *FOXP3* expression and Treg function during thymic differentiation, but it is not sufficient to isolate these cells. More recently, CD4^+^CD25^+^ CD127^-/lo^ (IL-7R α chain) phenotype has been used for isolation and identification of Tregs (29–32).

During Treg thymic differentiation, *FOXP3* expression depends on the coordination of several factors, including T cell receptor (TCR) signaling, CD28 co-stimulation (78), cytokines (IL-2, IL-15, and IL-7), transcription factors (NFAT and ICOS) (79, 80), and the PI3K-mTOR signaling network (81). Notably, *FOXP3* can also be expressed in differentiating pTregs or in iTregs upon TCR stimulation with suboptimal co-stimulatory molecules. However, transient expression of *FOXP3* in Teff cells did not correlate with regulatory functions previously reported in Tregs, indicating that *FOXP3* may not be used as a marker solely for Tregs (82). TGF-β and IL-2 stimulation coupled with TCR signaling and co-stimulatory molecules skew the differentiation of naïve CD4^+^ T cells into Tregs. Mechanistically, IL-2 triggers the STAT5 signaling network and its downstream targets, including the expression of *FOXP3*, and polarizes CD4^+^ T cell differentiation to Tregs rather than IL-17-producing effector T cells (Th17) (83, 84). Although human TGF-β-induced Tregs have a suppressive function *in vitro*, the transcriptomic landscape does not recapitulate tTregs, and its suppressive capacity is compromised *in vivo* in humanized GvHD mouse models (85).

**Figure 1:**
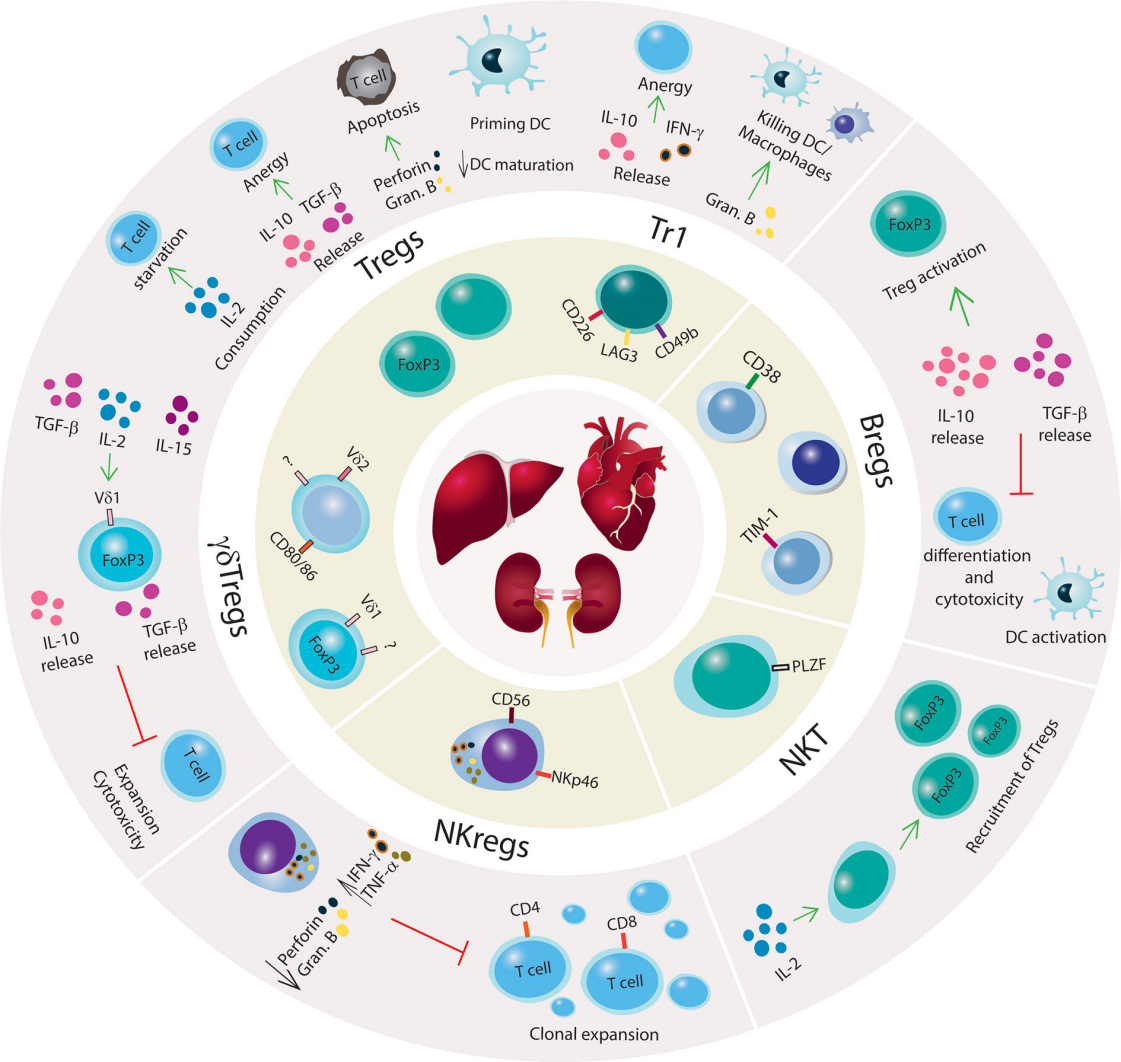
Mechanisms of immune tolerance to promote SOT engraftment and survival. Schematic illustration of regulatory innate and adaptive immune cells with a brief summary of mechanisms of immune suppression. Kidney, liver and heart are represented in the center of the figure, and regulatory immune cells (Tregs, Tr1, Bregs, NKT cells, NKregs, γδTregs) are shown surrounding the organs. The outer circle illustrates the main regulatory networks for each immune cell subset. Green arrows indicate promoting mechanisms, black arrows denote increase or decrease of cytokines production or biological processes, and red lines denote inhibitory networks.

Tregs can suppress autoimmunity directly through the release of cytokines (e.g. IL-10, IL-35, and TGF-β) or mediate cytotoxicity toward Teff *via* the production of proteases that induce cell apoptosis, such as granzyme and perforin, or galectins (86–91). Indirect mechanisms of suppression include: 1. recruitment of other cells, such as modulating antigen presenting cell (APC) function through cytotoxic T-lymphocyte antigen 4 (CTLA4), 2. expression of CD39/CD73 ectonucleotidases that convert ATP to immunosuppressive metabolites such as AMP and adenosine, 3. shifting a proinflammatory environment to anti-inflammatory (92, 93), and 4. outcompeting Teffs in IL-2 uptake by overexpressing CD25 (94) (**Figure 1**).

In the allogeneic transplant context, Treg signaling mechanisms are crucial for allograft survival because of their dampening of the immune response from Teff cells. As Tregs do not produce IL-2, their activation depends on the release of IL-2 by Teff cells (95). In the absence of Tregs, the binding of Teff TCR to alloantigen-major histocompatibility complex (MHC) and CD28 to CD80/CD86 activates Teff cells, leading to the secretion of IL-2 (86, 96). By autocrine mechanisms, IL-2 signaling triggers other T cells, causing activation, proliferation, and differentiation that can all lead to allograft rejection. However, activated Tregs secrete IL-10 and TGF-β that convert Teff cells into anergic cells, creating a tolerogenic environment. The expression of the co-stimulatory molecule CTLA4 on Tregs interacts with CD80/86 on dendritic cells (DCs) to suppress the immune response and contribute to allograft tolerance (97, 98).

Given their involvement in a multitude of immune responses, Tregs are considered a heterogeneous population with diverse functions and markers. Most of these cells can be categorized as naïve, *FOXP3* ^+^ effector non-Treg cells, or activated/effector Tregs with the latter being the most proliferative (Ki67^+^) and suppressive (CTLA4^high^) (33). Moreover, *FOXP3* ^+^ effector non-Treg cells produce pro-inflammatory cytokines such as IFN-*γ* and IL-7 that have reduced immunosuppressive function and a high potential to become Teff. A further subset of human effector Tregs was identified using chemokine receptors and intracellular markers wherein T helper-like Tregs showed a memory-like phenotype (36). The migratory capacity (**Table 1**) and cytokine secretion of each subset offers crucial information in the graft tolerance context given that these cells have the ability to target specific tissue types, such as allografts or lymph nodes (99).

### Tr1 Cells

In contrast to tTregs, regulatory type 1 T cells (Tr1) are a subpopulation of memory CD4^+^ T cells that can transiently express *FOXP3* upon activation; however, *FOXP3* expression in Tr1 cells is not constitutive or a requirement for Tr1 function and differentiation (100). Tr1 cells co-express integrin α2 subunit (CD49b) and lymphocyte activation gene 3 (LAG-3), which facilitate the identification of Tr1 in the peripheral blood of tolerant patients (39). LAG-3 is mostly expressed on activated Tr1 cells while CD49b expression is constitutive. Other cell surface markers have been identified including Tim-3, PD-1, TIGIT, and CD39, but they are not exclusive to Tr1 cells (101). Another crucial difference between Tr1 and tTregs is the metabolic profile on which these cells rely; Tr1 cells depend on aerobic glycolysis (102) while *FOXP3* ^+^ Treg differentiation is associated with fatty acid oxidative phosphorylation (103).

Tr1 cell development, expansion, and function are independent of IL-2 and CD28 (104). The Tr1 cell mechanism of suppression is *via* secretion of TGF-β and IL-10 in which IL-10 constitutively triggers Tr1 cells to release additional IL-10, creating a feedback loop (**Figure 1**). In the absence of IL-10, Tr1 cells lose their capacity to produce IL-10 but retain secondary mechanisms of immune suppression that are driven by the expression of granzyme B and CTLA-4 (105). Tr1 can release IFN-γ but only low or absent levels of IL-2, IL-4, and IL-17 have been found in these cells. The activation of Tr1 cells is *via* cognate antigen binding by their TCR, which initiates the production of granzyme B and Tr1-mediated killing of DCs or macrophages (40, 106). Once activated, Tr1 cells perform bystander suppression. Tr1 cells also utilize suppressive mechanisms that are shared with *FOXP3* ^+^ Tregs including interactions of co-stimulatory molecules CTLA-4 with CD80 and PD-1 with PD-L1 (107).

IL-10 is essential for Tr1 cell function in humans and mice, but the signaling mechanism has not been fully elucidated. The STAT pathway has been suggested as the downstream target of IL-10 signaling in Tr1 cells. Studies have shown that STAT3 interacts with proteins associated with a glycolytic metabolic environment that favors Tr1 cell differentiation (102). High activation of STAT3 in T cells induces Tr1 differentiation (108), and the induction of IL-10 is STAT1- and STAT3-mediated (109). IL-27 has been described in mice and humans as a critical cytokine that promotes IL-10 secretion and Tr1 differentiation (110, 111). Mechanistically, IL-27 triggers STAT3, which activates B lymphocyte-induced maturation protein-1 expression (112). Under specific conditions, IL-27 also triggers c-Maf and AhR transcription factors to activate IL-10 transcription such that AhR also contributes to granzyme B expression in Tr1 cells (111, 113, 114). Other transcription factors necessary for IL-27-mediated induction of Tr1 include BAFT, IRF1, and ITK (115, 116).

### Single Cell Strategies to Identify Tregs and Tr1 Subpopulations

Previous studies have pioneered the investigation of cell surface markers to identify subsets of regulatory T cells. Given advances in single-cell strategies, the heterogeneity of regulatory T cells has been reflected in the stratification of these cells according to novel cell surface markers, intracellular markers, and transcriptomic signatures (34, 37, 38, 117, 118). For example, in a human T cell atlas study using single-cell RNA-seq, new evidence was reported for regulatory T cell ontogeny indicating that, in fact, there are two populations of Treg progenitors with specific transcriptional signatures in the human thymus (118). The cell population called Treg_(diff)_ showed lower expression of *FOXP3* and *CTLA4* when compared to conventional Tregs. Another cell subset had features similar to Treg_(diff)_ but not to Tregs. This population was referred to as T_agonist_ and presented low *FOXP3* expression but high expression of a non-coding RNA (*MIR155HG*). The definition of these two populations opens new paths to investigate the functional roles of these recently identified progenitors and the mechanisms that skew Treg development to one progenitor or another. By understanding these features, it will be possible to improve the understanding of the post-transplantation scenario when Tregs from transplanted CD34^+^ are differentiated in the thymus.

Human Tregs and Teffs from peripheral blood as well as from mouse *Foxp3* ^GFP^ lymphoid organs were sorted and analyzed in a scRNA-seq screening (117). In both species, a similar transcriptomic profile (*FOXP3, IL2Ra, IL2Rb, IKZF2, TNFRSF1B)* was shown to distinguish Tregs from Teffs using an expression profile associated with cell ontogenesis, cell function, and metabolic processes. Notably, the intensity of TCR signaling strongly influenced the clusters of Treg cells, suggesting multiple differentiation states in the Treg pool (117). However, approximately 55% of the human Treg cell cluster overlapped with Teffs, indicating that *FOXP3* ^+^Tregs with a CD4^+^CD25^+^CD127^lo^ phenotype comprise a heterogeneous population with certain cells expressing an effector transcriptomic profile. In a scRNA-seq analysis of CD4^+^ T cells from pancreatic intragrafts of mice treated with CD47 monoclonal antibodies (mAb), two subpopulations of Tregs with low proliferative capacity and a distinct transcriptomic network were identified in rejected grafts (119). These results indicate that Treg heterogeneity is susceptible to changes in the microenvironment caused by, for instance, mAbs.

In a single-cell mass cytometry (CyTOF) study, human peripheral blood mononuclear cells (PBMCs) and isolated CD4^+^ T cells were analyzed for their cell surface and intracellular markers. Unsupervised high-dimension clustering analysis identified new subsets, their phenotypes, and the relationship among these cell subpopulations (37). In another single-cell CyTOF analysis of liver-transplanted children, a panel with 22 markers identified a remarkable enrichment for non-classic Tregs (CD4^+^ CD5^+^ CD25^+^ CD38^-/lo^ CD45RA^-^) in tolerant recipients compared to patients under IS (34). Specifically, CD5 has been shown as a marker to promote extrathymic Treg development in response to self or tolerizing agents in the periphery (120–122), while lack of CD45RA indicates a memory phenotype in kidney transplanted patients (123). This shows that these induced Tregs in the periphery can have high plasticity to immune responses (120, 122) and be generated in a tolerogenic environment. The identification of new cell subtypes in tolerant patients can define novel diagnostic markers that will benefit other SOTs.

Similarly, CyTOF analysis of sorted Tregs from healthy donors showed a heterogeneous population of naïve Tregs that failed to express markers commonly reported for conventional Tregs such as CCR4, CD39, HLA-DR, ICOS, and CD147 (38). Interestingly, hierarchical analysis of naïve Tregs using CD31, CD103, and LAP markers showed subpopulations carrying a preprogrammed status, suggesting a transient state between naïve and fully differentiated Tregs. Thus, the heterogeneous population of Tregs identified in tolerogenic liver-transplanted recipients may have transient states that can contribute to prolonged graft survival. Current studies in lineage tracing and pseudotime analysis of single-cell data will provide valuable information about the biological trajectory for regulatory T cell specification.

Recently, Miragaia et al. (124) compared Tregs from murine and human non-lymphoid tissues to identify a conserved transcriptional signature for peripheral Tregs that have travelled across tissues. Two subpopulations of transient Tregs were found with tissue-specific gene signatures that had adapted toward either skin or colon tissues (124). Many factors have been previously reported to induce, maintain, and attract Tregs to the colon, including dietary antigens and the microbiota (125). Tr1 cells have also been reported in gut-related autoimmune disorders. Tr1 cells generated and expanded *in vitro* specific for ovalbumin (OVA-specific Tr1) have been previously tested in Crohn’s disease and colitis (106, 126). In a clinical trial with OVA-specific Tr1 clones, patients ingested OVA-enriched diets to stimulate OVA-specific Tr1 cell migration to the gut. This study reported a decrease in tissue inflammation up to five weeks post-treatment and OVA-specific Tr1 immunoregulatory function *ex vivo.* Moreover, Tr1 cells induced *in vitro* can express gut-homing markers GPR15 and CCR9 (41), and Tr1 cells induced *in vivo* have been found in tolerant mouse models (39), indicating the migratory capacity of Tr1 cells.

Previous reports have extensively discussed therapies using Tregs and Tr1 to control and prevent GvHD (127, 128). The understanding of key molecular features in Tregs and Tr1 will improve therapeutic approaches and clinical protocols to mitigate GvHD and promote allograft survival. Results from more recent cutting-edge technologies will provide new insights into T regulatory networks, cell function, cellular states and plasticity, cell migration markers, and cell expansion and survival. Altogether, these studies highlight the critical importance of taking precautions in expanding Tregs based on specific phenotypes because these cells can carry subpopulations with effector function that may negatively impact allograft survival and function.

### Regulatory B Cells

Recent studies have identified potentially important contributions to tolerogenesis from humoral immunity, a section of the adaptive immune response. B cells are at the core of humoral immunity and are responsible for clonally producing antibodies, but immune regulatory function is less understood for B cells than for Tregs. In the bone marrow, various cytokines, chemokines, and transcription factors regulate B cell differentiation from hematopoietic stem cells. Premature B cells travel from the bone marrow to the spleen and secondary lymphoid tissues to mature and differentiate under antigen-dependent and independent phases of selection. Similar to T cells, B cell receptor (BCR) is expressed *via* V(D)J rearrangement during maturation and selection. The combination of the antigen recognition by BCR and a co-stimulatory signal (e.g., helper T cell binding) stimulates B cell proliferation into either plasma cells responsible for secreting antibodies or memory cells that have a high survival rate, high antigen affinity, and fast secondary response.

Studies have previously reported that the regulation of humoral immunity through either conventional mechanisms of immune suppression or B cell immunomodulatory functions can be crucial for the success of allograft transplant (**Figure 1**). Early evidence was found in 1970 when, upon B cell depletion, guinea pigs suffered severe and prolonged contact hypersensitivity responses, indicating a suppressive role of B cells toward T cell responses (129, 130). B regulatory cells (Bregs) have mostly been characterized by their capacity to secrete IL-10 and TGF-β; which curtail T cell differentiation and cytotoxic function. Briefly, the mechanism wherein Bregs also modulate T and Natural Killer (NK) cell apoptosis is *via* the production of granzyme B and FasL (131, 132). In humans, Bregs are phenotypically subdivided into multiple subsets including transitional TIM-1^+^ cells expressing IL-10 and memory/mature (**Table 1**) (44–46). In studies with human kidney allografts, the imbalance of IL-10/TNF-α expression in Breg cells was correlated with kidney injury (44). Additionally, tolerant recipients with complete eradication of the IS regimen showed elevated numbers of naïve, memory, and total B cells, upregulation in co-stimulatory and inhibitory molecules, and a genomic signature toward tolerogenesis (133, 134).

## Role of Innate Immunity in Promoting Tolerance

Despite its fundamental role in immune defense, innate immunity can also involve regulatory functions. Specific subsets of cell types involved in innate immunity can contribute to graft tolerance or rejection after SOT.

### γδ T Cells With Regulatory Properties

The classic identification of T lymphocytes involves the expression of either αβ TCR or γδ TCR (γδ T cells), although pro-inflammatory T cells bearing both receptors have been identified in mice and humans (135). Compared to αβ T cells, γδ T cells have less variability in the V and J gene segments, but γδ TCR have vast variation in the rearrangement of the D genes. Although other subsets have been identified within the γδ T population, Vδ1 and Vδ2 T cells remain the most studied subtypes. In humans, peripheral γδ T cells comprise up to 5% of the T cell population with Vδ2 as the major subset, but γδ T cells can rapidly expand in response to viral infections like human cytomegalovirus (CMV), inflammation, and tumors. Although Vδ1 T cells exist in the blood, they predominantly reside in the mucosal epithelia of solid tissues including the liver, skin, and intestines.

In comparison to αβ T cells, γδ T cells directly recognize antigens independent of MHC haplotype. The Vδ1 TCR binds to stress-induced proteins, such as MHC-I related chain A or B which are often found on tumorigenic cells and in post-SOT biopsies. The Vδ2 TCR recognizes small non-peptide phosphorylated antigens (pAg), which are intermediates of the mevalonate pathway in eukaryotes and in the non-mevalonate pathways in prokaryotes. For example, isopentenyl pyrophosphate (IPP) can accumulate in tumor cells carrying a defective mevalonate pathway. Mechanistically, members of the butyrophilin receptor family (e.g. BTN3A1) in either APC or tumor cells bind to IPP *via* intracellular domains and undergo conformational changes in the extracellular domains that are recognized by γδ TCR, leading to the activation of Vδ2 T cells (136). Recently, pAg-mediated coupling of BTN2A1 and BTN3A1 was suggested as the stimulatory trigger of Vδ2 T cells (137). Notably, γδ T cell subsets can recognize antigens *via* the expression of receptors commonly found on NK cells, such as NKG2D, DNAM-1, NKp30, and NKp44 (138).

Besides their anti-tumorigenic and anti-infectious role, γδ T cells can exert immune suppressive functions. In 1989, Patel et al. (139) reported regulatory properties of a specific subset of γδ T cells (γδ Tregs) involved in inhibiting mitomycin-activated CD4^+^ T cell to activate B cell maturation *in vitro*. The phenotypic identification of γδ Tregs has mostly been based on findings from functional assays *in vitro* and expression of markers previously reported for conventional Tregs. Peripheral γδ T cells from healthy donors have no detectable levels of *FOXP3* but show low expression of CD25 and CTLA-4. However, under IL-2, IL-15, and/or TGF-β stimulation, γδ Tregs can express *FOXP3*, release IL-10 and TGF-β; and inhibit the effector function of previously activated CD4^+^ T cells (47–51) (**Figure 1**). Recently, γδ Tregs expressing CD73 that secrete IL-10 and TGF-β were identified in both the periphery and tumors of patients diagnosed with advanced metastatic breast cancer (140).

The expression of co-stimulatory molecules (e.g. CD80, CD86) and inhibitory molecules (PD-L1) on Vδ2 T cells and results from transwell assays have provided evidence of the cell-to-cell contact dependency for γδ Tregs immune suppressive function (48). However, no consensus has been achieved regarding the cell culture method to expand and activate regulatory mechanisms in γδ T cells. The variations include γδ T isolation strategies prior to or after cytokine stimulation, different types of cytokines stimulation, co-culture with either PBMCs (without the removal of conventional Tregs pool) or selectively activated CD4^+^ T cells, anti-TCR γδ for activation, and presence or absence of pAgs (52). Although the largest population of γδ T cells carrying regulatory features are Vδ1 T cells, few studies have compared the immune regulatory function of Vδ1 and Vδ2 T subsets, and a comprehensive analysis of the suppressive capacity of γδ T subsets has not been clarified.

γδ T cells reside in several tissues where they can exert immune suppressive functions. For example, patients with active celiac disease had reduced levels of TGF-β-expressing γδ T cells, but patients on a gluten-free diet benefited from γδ Treg expansion and abrogation of Teff response (141). Additionally, the expansion of peripheral Vδ1 T cells in pregnant women and the production of IL-10 and TGF-β by γδ T cells in the uterus can promote a suppressive environment that is likely necessary for fetal-maternal interface to avoid rejection early in pregnancy (142, 143).

### NK and NKT Cells

Besides γδ T cells, NK and natural killer T (NKT) cells compose innate immunity. NK cells are known for exterminating tumor and virus-infected cells, and the term NKT cells derives from these cells’ similarities with both NK and T cells. Like NK cells, NKT cells express surface markers such as CD161. Like T cells, NKT cells differentiate and mature in the thymus and, phenotypically, can be CD4^+^, CD8^+^, or CD4^-^CD8^-^. Although CD4^-^CD8^-^ is indicative of immature T cells, activated NKTs with this phenotype are fully competent to produce cytokines (IL-4 and IFN-*γ*). In mice and humans, another marker shared among NK, NKT, T, and γδ T cells is promyelocytic leukemia zinc finger (PLZF). In mice, PLZF, together with GATA-3, ROR*γ*T, and T-bet, can stratify subpopulations of thymic NKT cells (144). Notably, NKTs have limited diversity in αβ TCRs, especially in humans (Vα24Jα18); NKT cells are activated when NKT TCRs detect glycolipid Ags presented by CD1d molecules on APCs. In mice, subpopulations of NKT cells in different maturation stages have been identified by the expression of NK1.1 (145, 146).

Although some NKT permanently localize to the thymus (147), a subset migrates to other tissues. The largest accumulation is in the liver where these cells make up approximately 30% of the T lymphocyte population (148). NKT subpopulations sensitive to IL-15 and positive for the transcription factor T-bet express chemokine receptors (e.g. CXCR3 and CXCR6) that bind to ligands produced in the liver (e.g., CXCL9, CXCL16) (149). In an IL-2-dependent manner, NKT cells recruit and trigger Tregs to tissues (**Figure 1**), indicating a regulatory function for NKTs that is also crucial for tolerance in coupled stem cell and solid organ transplants (150). Recently, Zhou et al. (151) focused on single-cell analysis of human peripheric NKT cells to characterize the transcriptomic signatures in NKT subpopulations. By evaluating the gene expression of specific cytokines, one NKT subset showed an immune regulatory profile comprising IL-2^+^, IL-10^+^, ICOS^+^, IL-4^–^, IFN-γ^–^, and XCL^–^. In cancer studies, a CD4^+^ NKT population was reported with immune modulatory function (152, 153). In the context of allogeneic HSCT, low levels of CD4^+^ NKT cells were correlated with the development of chronic GvHD in patients that received grafts from BMT (154).

NK cells distinguish between autologous and allogeneic cells *via* inhibitory receptors present on the cell surface that identify self-antigens and prevent cell lysis. For example, at later stages of maturation, NK cells express killer immunoglobulin-like receptors (KIRs) that bind to classic MHC-I. A NK tolerogenic marker is the heterodimer CD94/NKG2A that specifically recognizes HLA-E and is expressed at the early stages of NK differentiation (155). KIRs and CD94/NKG2A can be co-expressed at intermediary stages of differentiation, but to avoid autoreactivity, mature NKs selectively express one or the other (156). In humans, the receptor NKG2D recognizes stress-related ligands MICA and MICB, triggering NK cell toxicity (157). However, NKG2D is not exclusive to NKs as it is also expressed by γδ T cells and NKT cells. Other cytotoxic-related receptors found in NK are Nkp30 and Nkp46 (158).

As in other immune subsets, human NKs are heterogeneous with subpopulations mostly distinguished by different expression levels of CD56 and CD16. Terminally mature NKs with a cytotoxic phenotype are CD56^dim^CD16^+^ and are the vast majority of circulating NKs in the periphery. These mature NKs also have higher expression of KIR or CD94/NKG2A. In two single-cell transcriptomic analyses of NK cells from peripheral blood and bone marrow of healthy donors (159, 160), CD56^dim^CD16^+^ were reported as heterogeneous with only one subset (also CD57^+^) showing a singular transcriptomic profile of terminally different NKs (high expression of *CX3CR1*, *TIM-3*, and *ZEB-2)*. Conversely, CD56^bright^CD16^-/low^ have an immature state and express NKG2A, but KIR is absent in these cells. In pseudotime trajectory analysis to determine lineage specification, CD56^bright^CD16^-/low^ were found as precursors of CD56^dim^CD16^+^ based on their transcriptomic profile (160). A transitional state between immature and terminal NKs was also reported and indicated the following developmental trace: CD56^bright^CD16^-/low^ cells to CD56^dim^ CD57^-^ and then CD56^dim^ CD16^+^ CD57^+^.

The subset of CD56^bright^CD16^-/low^ cells secrete IFN-*γ* and TNF-α but express low to no levels of perforin and granzyme B, indicating a regulatory profile (CD56^bright^ NKreg) rather than a cytolytic role (**Figure 1**). However, prolonged stimulation with IL-2 and IL-5 can activate CD56^bright^ cells to become cytolytic and differentiate into CD56^dim^ in a mechanism mediated by the STAT3 signaling network (161). CD56^bright^ NK cells have been identified in an immune suppressive environment, such as in the uterus and periphery of pregnant women, leading to high response against viral infections and tumorigenesis as well as positively affecting successful full-term pregnancies (162, 163).

## Therapeutic Strategies to Improve Immune Cell Tolerance

While immunosuppression has contributed to substantial improvements in graft survival in SOT, investigators have recognized the need for other mechanisms to promote transplant tolerance in order to avoid the implications of long-term IS administration. The combination of HSCT and SOT is an important development, but numerous challenges remain in optimizing graft survival without GvHD, excess risk of infection, or lifelong need for IS drugs. Applying the growing understanding of cells involved in immune tolerance can improve HSCT and SOT as a therapeutic strategy and lead to enhanced long-term patient outcomes.

### Tolerance in HSCT and SOT

Since the early 90s, Strober and collaborators have sought to develop the combination of haplo-HSCT with kidney transplant. Chimerism, the coexistence of both donor and recipient hematopoietic cells, is a critical mechanism for promoting tolerance in this approach. Chimerism that persists for at least six months after transplant is associated with improved kidney graft tolerance and effective immune response to infection (28, 164). Both HLA-matched and mismatched HSCT with SOT can achieve chimerism, but persistent chimerism that is believed to promote tolerance has been achieved more frequently in HLA-matched recipients (25).

Busque et al. (25) reported that 24 of 29 HLA-matched transplant recipients with stable mixed chimerism for at least 6 months were able to discontinue IS drugs within 6-14 months with no cases of GvHD and only one case of graft loss. Ten patients had mixed chimerism that persisted after cessation of IS drugs. The remaining patients lost mixed chimerism without IS, but only one experienced graft rejection (2, 25, 165, 166), suggesting that durable operational tolerance may be induced by prior mixed chimerism (28).

In contrast, HLA-mismatched HSCT and kidney transplant recipients have typically needed chronic IS drugs to avoid graft rejection and GvHD (26, 27, 165). HLA-mismatched patients with mixed chimerism 12 months post-transplant were able to taper to one IS drug (tacrolimus), but full cessation resulted in loss of chimerism and evidence of graft rejection that required reinstatement of single-agent tacrolimus. HLA-mismatched patients who do not develop mixed chimerism that lasts beyond three weeks after transplant were prone to engraftment syndrome and associated graft injury that occurred despite continued IS (25). Another approach in HLA-mismatched kidney transplants has been to induce tolerance with full rather than mixed chimerism. While this approach enabled 22 of 37 patients to discontinue IS therapy, there were two cases of GvHD, one of which was fatal and the other chronic (25, 28, 167). Side effects of an intensive conditioning regimen in this approach led to severe neutropenia and thrombocytopenia post-transplant, and two patients experienced graft loss due to infection (25, 28).

Multiple therapeutic strategies to avoid these difficult tradeoffs have been proposed and are being evaluated in preclinical and clinical studies. A promising approach involves HSCT graft engineering that capitalizes on a deepening understanding of regulatory cells to cultivate tolerance independent of IS drugs without GvHD or excess infection risk.

### Graft Manipulation to Optimize Sequential HSCT and SOT

In the context of hematologic malignancies, HSCT graft manipulation techniques have shown clear benefits, and many of these approaches could be applied and enhanced to improve combined HSCT and SOT. A breakthrough approach in hematologic diseases reported that using G-CSF to mobilize HSCs and hematopoietic stem cell progenitors (CD34^+^) from the bone marrow of the donor allows infusion of more CD34^+^ cells. Subsequent studies selectively depleted T cells *ex vivo* for obtaining a CD34^+^-enriched graft (>10×10^6^ cells/kg) and reported successful and prolonged engraftment in more than 90% of adult patients (168). However, slow immune reconstitution due to lymphocyte absence in the graft increased the susceptibility of these patients to lethal infection. With the discovery of Tregs and of their translational application, grafts enriched for CD34^+^ and co-infused with Tregs with a fraction of conventional T cells were infused in 43 conditioned patients with acute leukemia (169). These patients received no subsequent IS and had successful engraftment, but 15% developed acute GvHD, likely from the Teff cells in the graft.

In 2010, our group pioneered αβhaplo-HSCT (170), a new approach that eliminates the αβ T cells and CD19^+^ B cells from the graft. By removing the T cell subsets responsible for GvHD, this graft manipulation approach dramatically reduces the risk of severe acute and chronic GvHD (170, 171). Another benefit of this strategy is the presence of NK and γδ T cells in the graft that can immediately respond against infections, reducing patients’ morbidity and mortality. In fact, despite the removal of αβ T cells, the presence of mature donor-derived effector cells provides anti-infectious control while minimizing the risk of severe acute GvHD (172, 173). In both malignant and non-malignant disorders, αβhaplo-HSCT recipients have experienced excellent clinical outcomes including rapid immune reconstitution, low risk of infections, and low incidence of graft failure (170, 171, 174–176). As a result, αβhaplo-HSCT represents a potentially ideal approach for inducing a tolerogenic environment that enables successful SOT (177).

### Regulatory T Cells

Encouraging preclinical and clinical studies of Treg and Tr1 cells in autoimmune and inflammatory diseases (178, 179) suggest that regulatory T cell infusion could improve outcomes of SOT. In 2016, Todo et al. (180) published data from the first clinical trial with Tregs and liver transplanted patients (**Table 2**). Seven patients showed signs of transplant tolerance and were weaned off IS drugs starting at 6 months after SOT with complete withdrawal within 18 months. However, the same strategy failed in kidney transplanted patients (186). Although the cells transferred to these patients also carried Teff cells, this clinical trial is considered the first pilot study in humans of a strategy to induce allograft tolerance using Treg infusion.

**Table 2:** Brief summary of ongoing or completed clinical trials combining immune cell infusion with solid organ transplantation.

Clinical trial name and/or ID	Phase	Cells infused	Concentration of cells infused	Organ transplanted	Time of cell infusion	Reported outcomes	References
UMIN-000015789	I/II	CD4^+^CD25^+^Foxp3^+^	23.30 ± 14.38 × 10^6^	Liver	13 days post-SOT	Positive signs of transplant tolerance	(180)
		Treg enriched cells				Complete withdrawal of IS within 18 months	
LITTMUS (NCT03577431)	I/II	Donor alloantigen reactive CD4^+^CD25^+^CD127^lo^ Treg	2.5-125 × 10^6^ cells	Liver	Combined with SOT	Ongoing	–
LITTMUS (NCT03654040)	I/II	Donor alloantigen reactive CD4^+^CD25^+^CD127^lo^ Treg	90-500 × 10^6^ cells	Liver	Combined with SOT	Ongoing	–
ARTEMIS (NCT02474199)	I/II	Donor alloantigen reactive Tregs	300-500 × 10^6^ cells	Liver	2-6 years post-SOT	Recently completed	–
dELTA (NCT02188719)	I/II	Donor alloantigen reactive Tregs	50 × 10^6^ cells	Liver	3 months post-SOT	Recently completed	–
TASK (NCT02088931)	I	Autologous polyclonal CD4^+^CD25^+^CD127^low^ Tregs	224-384 × 10^6^ cells	Kidney	6 months post-SOT	No negative reaction to infused Tregs	(181)
						No infections	
ONE Study (NCT02091232)	I	Tregs	–	Kidney	7 days post-SOT	Completed	(182)
ONE Study/ONETreg1 (NCT02129881)	I/II	Autologous Tregs	1-10 × 10^6^ cells/kg	Kidney	5 days post-SOT	Ongoing	–
ONE study/ONEnTreg13 (NCT02371434)	I/II	Autologous, polyclonally expanded CD4^+^CD25^+^Foxp3^+^	0.5 × 10^6^ cells/kg or 1 × 10^6^ cells/kg or 2.5-3 × 10^6^ cells/kg	Kidney	Post-SOT	No rejection	(183)
		Tregs				Tapering of Immunosuppression drug for >70% of the patients	
						No infections	
ONE Study/darTREGs (NCT02244801)	I	Donor alloantigen reactive Treg	300× 10^6^ cells/kg	Kidney	Post-SOT	No rejection	(182)
Trex001 Study (NCT03867617)	I/II	Autologous *in vitro* expanded Tregs (CD45RA^+^CD4^+^CD25^high^CD127^low/neg^)	0.3-1.5 × 10^6^ cells/kg	Kidney	3 days post-SOT	Ongoing	(184)
STEADFAST	I/IIa	Autologous Antigen-Specific CAR-Treg	25 × 10^6^ cells	Kidney	Post-SOT	Ongoing	–
TOL-1 (NCT02560220)	I	Peripheral Blood Mononuclear Cells (MICs)	1.5 × 10^6^ or 1.51× 10^8^ MICs/kg	Kidney	2 or 7 days before SOT	Persistent high frequencies of Bregs	(185)
						No rejection	

Building on this pilot study, investigators have started turning to modified strategies for therapeutic Treg infusions. Expansion of human Tregs for clinical applications opened opportunities for the treatment of unwanted immune responses such as in autoimmunity and after transplantation. The identification of markers for subpopulations of Tregs (187) is allowing the isolation and removal of non-Tregs from the remaining Treg populations as part of cellular therapies for allograft tolerance. Additionally, manipulation of specific subsets of Treg effector cells may enable refining their immune suppressive functions (188, 189).

The advances in next generation sequencing-based strategies have been extended to evaluating TCR repertoire diversity and antigen specificity (190). T cell populations have multiple TCR clones resulting from previous and current exposure to antigens. The understanding of the TCR composition reflects prior infections, immunizations, and individual response to specific epitopes. For the transplantation field, clinical trials have evaluated polyclonal and donor antigen reactive Tregs (**Table 2**) to determine their therapeutic ability to promote a tolerogenic environment. Although patients were still under an immunosuppressive regimen, the analysis of donor-specific TCR repertoire from Tregs cultured with activated donor B cells separated nontolerant from tolerant kidney-transplanted patients (191). Moreover, tracking donor-specific Tregs repertoire may provide insights into stratifying patients according to the likelihood of successfully withdrawing immunosuppression. Growing evidence suggests that disease-relevant and antigen-specific Tregs offer advantages over polyclonal Tregs (192, 193). Donor Tregs have demonstrated better suppressive function towards alloreactive effector T-cells when compared to polyclonal Tregs, which can affect the number and purity of infused cells (194, 195). While expanded CD4^+^CD25^+^ Tregs have been used in clinical trials (196) with promising results in preventing GvHD, they are polyclonal, nonspecific and could induce universal immunosuppression. As a result, ongoing or recently completed clinical trials are focusing on purifying Tregs with or without alloantigen specificity (**Table 2**; LITTMUS, ARTEMIS, dELTA).

To improve tolerance in SOT recipients, other investigators explored the role of transient mixed chimerism (26). Previous observations showed that mild conditioning regimens can induce transient chimerism and tolerance, but myelosuppression was still required (197–200). The Trex001 Study (**Table 2**) will test an immunotherapy strategy to induce transient chimerism while reducing myelosuppression to promote a tolerogenic environment and prevent kidney rejection (184).

In kidney transplant recipients (**Table 2**; TASK), results from follow-up biopsies after two weeks and six months post-Treg infusion showed that no patient had a negative reaction to the Tregs, and no infections were observed (181). Interestingly, circulating Tregs peaked two weeks post-infusion and then declined until untraceable three months post-infusion. The ONE Study (**Table 2**) is a multi-center consortium testing the safety and feasibility of multiple Treg infusion protocols in kidney transplant recipients. Although the immunosuppression regimen is consolidated among the centers (tacrolimus, mycophenolate, and steroids for three months), differences include the clonality, donor origin, frozen or fresh cells, and expansion with or without co-stimulation. In the ONEnTreg13 trial, the infused nTregs became oligoclonal over time, favoring specific TCR repertoires selectively to alloantigens and potentially helping a tolerant environment post-SOT (183). As the numbers of Tregs in circulation decreased after a month of infusion, the study hypothesized that these cells homed to the graft. Although the protocol of the multicenter ONE Study was considered safe, the infusion of nTregs was insufficient to completely remove the three IS drug treatment post-SOT (182). These results indicate the need for strategies to improve Treg cellular therapy.

Collectively, these completed and ongoing trials will offer valuable information about safety, therapeutic strategy, and the most suitable time point in which to infuse Tregs after SOT.

### Engineering Tregs

Gene therapy to engineer Tregs offers another intriguing approach for HSCT and SOT, and major advances in designing Treg cell therapies and various gene editing methods are comprehensively discussed by Ferreira et al. (201).

Antigen-specificity in regulatory T cells could be obtained through the TCR or gene transduction of a chimeric antigen receptor (CAR). HLA-A mismatching is one of the critical factors affecting graft outcome; therefore, targeting HLA-A *via* antigen-specific Tregs may be a promising method of inducing tolerance (202, 203). MacDonald et al. (195) generated CAR Tregs expressing an HLA‐A2‐specific CAR (A2-CAR), which maintained Treg phenotypes and stability and could suppress CD8 T cell proliferation *in vitro*. They demonstrated that CAR Tregs were more potent than Tregs expressing an irrelevant CAR in preventing GvHD in a xenogeneic mouse model receiving HLA-A2^+^ human PBMC (195). Their results imply that the “off-target” effects of CAR-expressing Tregs is not different from the polyclonal Tregs. Nevertheless, Treg suppressive response is more likely to be induced *via* CAR than *via* TCR because it requires fewer target antigens. Moreover, the CAR Treg strategy allows for a lower number of Tregs that in turn decrease the off-target toxicity (195).

Dawson et al. (204) showed that the insertion of the wild type CD28 co-stimulatory domain is essential to the effective function of CAR Tregs *in vitro* and in an HLA-A2-mismatched xenoGvHD mouse model. Notably, RNA-seq analysis of CAR Tregs highlighted that stable expression of Helios and ability to suppress CD80 expression on DCs were major predictors of an effective *in vivo* performance (204). By incorporating in silico analysis, this comprehensive study showed that humanization of scFvs decreased cross-reactivity to several HLA-A allelic variants but could alter affinity and antigen specificity of CAR. This highlights the importance of testing multiple CARs to identify the optimal constructs. Determining allo-antigen specificity of Tregs is critical in the transplantation context to ensure precise targeting of allogeneic cells, tissues, and organs. Tregs expressing the optimal humanized A2-CARs showed rapid trafficking and persistence in HLA-A2-expressing allografts, migrated to draining lymph nodes, prevented HLA-A2^+^ cell-mediated xenogeneic GvHD, and effectively suppressed rejection of human HLA-A2^+^ skin allografts (205).

Adoptive transfer of A2-CAR Tregs was utilized to prevent rejection of human skin allograft in mice (202). A2-CAR Tregs potently suppressed the allogeneic responses of delayed-type hypersensitivity and prevented rejection of HLA-A2-positive human skin grafts for over 40 days, an effect attributed to A2-CAR Tregs homing to skin grafts and long-term persistence (202). In a similar study, CAR Tregs exhibited a greater suppressive function than ΔCAR Tregs (lacking CD28-CD3ζ domain) or polyclonal Tregs *in vitro* and ameliorated the alloimmune‐mediated skin injury (203). These studies demonstrate that human CAR Tregs specific for HLA-A2 are more protective than polyclonal Tregs in humanized skin transplants. Altogether, these studies lay the foundation for developing HLA-specific CAR Tregs as adoptive cell therapy for autoimmune diseases and SOT.

To further extend CAR technology to Treg application in mice, Pierini et al. (206) showed that Tregs with transient expression of mAbCAR (engineered FITC-targeted-CARs activated with FITC-conjugated mAbs) promoted suppressive function once incubated with FITC-mAbs *in vitro* and *in vivo* and induced homing of mAbCAR Tregs to specific cells and organs (206) Adoptive transfer of mAbCAR Tregs reduced allograft responses such as GvHD, prolonged MHC-mismatched pancreatic islet allograft survival, and increased alloantigen-specific tolerance to secondary skin grafts (206). Although this strategy is promising, FITC could induce immunogenicity in humans, a limitation that can be resolved with the use of clinically safe antibody-tagged systems. Nevertheless, these findings highlight the flexibility of the mAbCAR Treg approach and suggest benefits in its application in transplantation to induce tolerance while controlling GvHD.

Although promising results were described with CAR Tregs in preclinical studies, there are several concerns surrounding the translation of these approaches to human HSCT and SOT. First, immune-deficient NSG mice lack the complexity of the human immune system, which may affect interpretation of data regarding tolerance and safety. Second, adoptively transferred CAR Tregs are only present at the initial phase after transplantation, which increases the chance of graft rejection (207). Third, obtaining clinically relevant numbers of CAR Tregs that can survive long-term in SOT patients is challenging. Indeed, IS drugs may reduce the number of CAR Tregs in liver and kidney transplants, which could impact CAR Treg efficacy. In 2019, Sangamo Therapeutics, Inc. (UK) started the STEADFAST clinical trial (**Table 2**) to evaluate CAR Treg therapy for the prevention of immune-mediated rejection following HLA-A2 mismatched kidney transplant in end-stage renal disease. This trial will soon provide information about the short-term safety and tolerability of CAR-Tregs as well as insights into the impact of CAR Tregs that can be incorporated into future SOT clinical trials.

### B Cell Strategies

In the allograft context, antibody-mediated rejection (AMR) is a leading cause of graft loss (208, 209). The activation of long-lived plasma cells and B cells releases donor-specific antibodies (DSA) that bind to the endothelium of the allograft. This binding triggers the recruitment of NK cells, neutrophils, and macrophages, leading to a series of inflammatory events, cytotoxicity, and cellular necrosis (210, 211). The outcome is severe endothelial injury, platelet aggregation, thrombotic microangiopathy, and the eventual loss of allograft function. For this reason, influencing B cell activity to facilitate tolerance can directly influence the success of SOT.

Importantly, the choice of initial IS regimen can influence the overall differentiation profile of B cells (212). This, in turn, can impact the variety and quantity of specific Bregs after SOT. For example, sirolimus significantly expanded Bregs and *FOXP3* ^+^ Tregs one month after liver transplant (213), but this effect was not observed for tacrolimus. Transcriptomic studies coupled with flow cytometry analysis have shown that Bregs express inhibitory/co-stimulatory molecules, such as PD-L1, CTLA-4/CD80, and CD86 (214–216), known to promote Treg function including dampening of Teff response. Although treatment with belatacept, a CTLA-4-immunoglobulin fusion protein, was first developed to target T cells, low levels of BAFF were detected in tolerant patients (217). The results from a 10-year follow-up trial showed that the numbers of Breg cells as well as *FOXP3* ^+^ Tregs were constitutively elevated in patients treated with belatacept (218). This provides evidence that the combination of strategies targeting multiple levels of immunosuppression can benefit transplant recipients.

In a phase I clinical trial, modified immune cells (MICs) were stimulated with the alkylating agent mitomycin C, resulting in immature donor-derived DCs with high immune modulatory capacity (**Table 2**; TOL-1) (185). Although these patients were under steroid regimen, circulating Bregs were present in high numbers one month post-transplant with a persistent and significant increase in Breg frequencies two years later. The allograft function was normal, and, as the patients showed unmodified levels of Tregs compared to pretransplant and pretreatment levels, the effectiveness of the treatment could be associated with the tolerogenic capacity of Bregs. Other therapeutic strategies targeting molecular regulators of Breg function, such as TIM-1, histone deacetylase, and the STAT3 network pathway, can have meaningful impact in inducing humoral-mediated immune suppression in tolerant allograft recipients (219).

Therapies with mAbs in kidney, liver, and heart recipients have shown efficacy in mitigating graft loss and improving long-term outcomes. In renal-transplant patients, *de novo* and increased pre-formed DSA correlated with high levels of the B cell survival factor, BAFF, and elevated rates of AMR (220). Concordantly, in a phase II clinical trial, treatment with an anti-BAFF mAb (belimumab) after SOT reduced the formation of *de novo* DSA, dampened the number of active memory B cells, and expanded Breg cells (221). In another study, treatment with alemtuzumab, an anti-CD25 antibody, correlated with good clinical outcomes including expansion of transitional Bregs one year after kidney transplant (222). In heart allograft recipients, a single dose of rituximab pre-transplant was sufficient to support B cell differentiation, but a second dose at 15 days post-transplant accelerated graft rejection and led to poor outcomes (223). Taken together, these results indicate that clinical protocols using mAbs can modulate the humoral response with potential benefits in transplant recipients, and further studies will provide optimization of drug choice, dosage, and timing.

### γδ T Cell Strategies

Preclinical and observational studies have reported γδ Treg function in SOT survival and homeostasis. In mouse models of kidney and liver transplant, the enrichment of peripheral CD8^+^ γδ T cells was positively correlated with graft tolerance as these cells secreted suppressive cytokines (e.g. IL-10 and IL-4) toward Th1 responses (53). Moreover, IL-4 dampened Vδ2 T cell function and increased the IL-10-secreting Vδ1 T cell population (224).

The ability of γδ T cells to control viral infections is important in the transplantation setting as HCMV infection is a major complication in transplant recipients. Interestingly, enrichment of cytotoxic Vδ1 T cells with an effector memory phenotype has been found in αβhaplo-HSCT (225) and kidney recipients (226) positive for CMV. In fact, HCMV reactivation was resolved one year after kidney transplant in patients with elevated Vδ2^-^ T cells. To amplify the benefits of γδ T cells, Vδ2 T cells from αβhaplo-HSCT recipients can be expanded under zoledronic acid (Zol) treatment *in vitro* whereupon these cells show an effector memory phenotype and aggressive cytolytic capacity against leukemia cells (225). In 43 pediatric leukemia patients transplanted with αβhaplo-HSCT, multiple Zol infusions were safe and improved overall survival, potentially due to the promotion of strong cytotoxicity against leukemia cells from Vδ2 T cells (227). In previous studies, Zol-activated Vδ2 T cells infused in patients with solid tumors reestablished a γδ T cell reservoir and halted cancer progression (228). Notably, γδ Treg cells can be induced *in vitro* under Concanavalin A treatment (47), indicating its potential function for γδ Treg expansion *ex vivo.*

In pediatric liver transplant recipients, the increase in Vδ1/Vδ2 could indicate successful long-term tolerance (229). Reduced incidence of GvHD was associated with increased levels of CD27^+^ Vδ1 T cells in patients who received allogeneic HSCT (230). This study also reported that G-CSF can significantly increase donor γδ Tregs *in vivo* and *in vitro*, suggesting that the choice of mobilization agent can influence the immunosuppressive environment of the graft. However, a clear phenotype to identify and isolate γδ Tregs is still under investigation, which limits the understanding of the molecular mechanisms of suppression and the appreciation of findings in clinical tolerance. Despite an absence in the literature regarding approaches to engineer or expand γδ Tregs for clinical applications, other recently developed strategies for genetic modifications of γδ T cells may propel future studies in γδ Tregs (231).

### NK and NKT Cell Strategies

In light of the importance of NK and NKT cells for both immune defense and tolerance, these cells have a potentially impactful role in successful transplant outcomes. NK and NKT cell infusions to prevent GvHD are under preclinical investigation (127). In previous studies of post-HSCT recipients, expansion of NKT cell subpopulations positively correlated with GvHD mitigation (232). CD56^bright^ NKreg cells positive for NKp46 have been found to be related to a low incidence of GvHD and have been used as a marker in clinical studies (58). In fact, NKp46 receptors are the drivers of NK response to eliminate HCMV-infected DCs. In clinical trials of chronic HCMV-infected patients who received liver transplants, IS administration was removed for half of the recipients (233). The tolerogenic environment was associated with the expansion of CD8^+^ T cell expressing regulatory markers (CTLA-4, TIM-3, PD-1) and the upregulation of genes downstream of IFN-*γ* signaling (ISG15, IRF1/7/9), suggesting an immune response that includes NK cell mechanisms. Low levels of non-cytolytic NKregs were associated with chronic GvHD 100 days post-HSCT in HLA-matched recipients enrolled in the ABLE/PBMTC1202 study (58, 59). An ongoing clinical trial (NCT03605953) is testing the feasibility of expanding and injecting donor CD4^-^ NKT cells post-allogeneic HSCT to promote graft versus leukemia (GvL) while reducing the risk of GvHD.

In the graft manipulation approach of αβhaplo-HSCT, donor NK cells (30-40 × 10^6^ per kg) were included in cell infusion for children with acute myeloid and acute lymphocytic leukemia (170, 176). To improve the clinical outcome in terms of GvHD and GvL, the infused NK cells were selectively chosen according to their alloreactivity based on KIR/KIR-ligand model, KIR B haplotype, size of NK alloreactive subset, and high expression of NKp46 and NKG2C (170, 234). While the NK alloreactivity was not observed to be crucial for the overall GvL effect, NK cells were believed to reduce GvHD and short-term infection risk.

NK cell alloreactivity plays a major role in SOT as the graft can be recognized by NKs under a “missing self” mechanism which potentially leads to graft rejection (235). KIR-HLA mismatch has been shown to negatively impact short- and long-term survival of kidney grafts (236). However, to date, no clinical consensus has been reached regarding the use of KIR-ligand as a predictive model for transplantation outcome because other NK receptors can also mediate alloreactivity and tolerance. Solid organ transplant recipients may also benefit from infused or recently differentiated NKreg cells. In other clinical studies, immature NK cells (CD56^bright^NKG2A^high^KIR^low^) derived from hematopoietic stem cell differentiation were identified in the first weeks post-HSCT (237–239). These results have important implications in the context of SOT as immature NK cells may carry an NKreg subpopulation that will offer a tolerogenic environment to improve graft survival. Moreover, understanding the peak of the noncytolytic NKregs pool, as well as of other regulatory cells, can offer the best timing of SOT post-HSCT. Further studies identifying NKT cell phenotype and function will provide valuable information for understanding the cytotoxic and regulatory role of NKTs subsets, especially in the context of HSCT and SOT.

In recent years, there has been substantial investigation to develop off-the-shelf products for cell therapy. Although the synthesis of CAR-NKs is challenging, CAR-NK cells have the potential to become universal therapies. Preclinical and clinical studies have shown promising safety and efficacy for CAR-NK in cancer immunotherapies and in reducing GvHD (240). To date, the feasibility of CAR NKreg tolerogenic potential for SOT has not been tested. Understanding the mechanisms and phenotypes of NKreg cells will enable development of targeting strategies using CAR or CRISPR-Cas9 gain-of-function to create a tolerogenic environment and reduce graft loss.

## Conclusion

The transplantation tolerance field has dramatically advanced over recent decades to improve organ engraftment and survival and abate the mortality and morbidity caused by IS. Despite major advances, widespread tolerance in SOT has not yet been achieved without dependence on IS regimens. Several preclinical studies have confirmed the feasibility for inducing transplantation tolerance; however, there remains a gap in translating these findings to the clinic.

One major challenge is represented by the variability in outcomes depending on the type of solid organ transplant. To prevent graft loss, immunosuppression regimens are proportional to the likelihood of graft rejection for specific organs. Allogeneic skin transplants are the most complex model of transplantation due to high immunogenicity and high numbers and varieties of APCs (241). Intestine transplants are also at high risk of rejection while heart, kidney, and liver transplants carry a lower risk. Given its function in metabolism and detoxification, the liver receives and processes large quantities of bacteria and dietary products and, accordingly, has a persistent, well-regulated immunoregulatory property. The benefit of low hepatic immunogenicity is to offer systemic immune tolerance and successful engraftment for a co-transplanted organ, such as liver and kidney co-transplants (242, 243). Mechanistically, liver-resident macrophages and hepatic myeloid and plasmacytoid DCs produce and secrete IL-10 and prostaglandins which reduce the expression of co-stimulatory receptors on APCs and compromise the activation of Teff cells (244–249). Myeloid populations provide additional regulatory mechanisms to prevent CD4^+^ T cell activation *via* IL-10, TGF-β; and IDO (250). In preclinical studies of kidney and heart transplants, host DCs rapidly replace donor DCs within days post-transplant and are associated with graft rejection (251–253). The depletion of graft DCs was reported to delay ongoing acute rejection. Thus, the diversity in immunogenicity across tissues poses a challenge in predicting the outcome of SOTs that apply similar transplantation strategies.

Despite these difficulties, pairing allogeneic HSCT with SOT is a promising approach. Besides dramatically increasing the chance of finding a suitable donor for the organ transplant, combining allogeneic HSCT with SOT can positively impact allograft survival and overall clinical outcomes. More recently, modifications in HSCT and SOT protocols have successfully decreased or eliminated IS administration for select patients. Further improvements are needed to consolidate and expand these results. In HSCT, graft manipulations, such as αβhaplo-HSCT, have successfully minimized IS administration while contributing to the prolonged survival of pediatric and adult patients (170, 174, 254). A deeper understanding of regulatory and suppressive immune mechanisms has vast applicability in inducing tolerance in transplant patients and bringing αβhaplo-HSCT and other techniques into SOT. In the era of single-cell data, novel regulatory subsets have been more comprehensively studied in their transcriptomic, epigenomic, and immunophenotypic profile, providing new avenues for amplifying immune tolerance. By recapitulating or increasing cellular regulatory networks, engineering strategies to manipulate immune cells *in vitro* for subsequent infusion can prolong tolerance post-HSCT which may offer a suitable window for SOT and allograft survival. Taken together, technological advances and ongoing clinical trials in these areas will appreciably change the field of transplantation tolerance.

## Author Contributions

PFS and AB outlined and wrote the manuscript. PFS designed the figure and the tables. MY reviewed the literature and wrote about engineered CAR-Tregs. PFS, MY and AB edited and approved the final version of this manuscript. All authors contributed to the article and approved the submitted version.

## Funding

This work was partially funded by the Kruzn For a Kure Foundation. PFS is funded by the Pediatric Nonmalignant Hematology and Stem Cell Biology NIH T32 training grant (DK098132).

## Conflict of Interest

The authors declare that the research was conducted in the absence of any commercial or financial relationships that could be construed as a potential conflict of interest.
